# Assessing inbred–hybrid relationships for developing drought‐tolerant provitamin A–quality protein maize hybrids

**DOI:** 10.1002/agj2.20344

**Published:** 2020-09-10

**Authors:** Ebenezer Obeng‐Bio, Baffour Badu‐Apraku, Beatrice Elohor Ifie, Agyemang Danquah, Essie T. Blay, Mustapha Abu Dadzie

**Affiliations:** ^1^ CSIR‐Crops Research Institute P.O. Box 3785, Fumesua Kumasi Ghana; ^2^ International Institute of Tropical Agriculture (IITA) PMB 5320, Oyo Rd Ibadan Nigeria; ^3^ West Africa Centre for Crop Improvement (WACCI) Univ. of Ghana PBM 30 Legon Accra Ghana; ^4^ Cocoa Research Institute of Ghana New Tafo‐Akim Ghana

## Abstract

Drought‐tolerant early‐maturing maize (*Zea mays* L.) inbred lines with high levels of provitamin A (PVA) and quality protein (QPM) are urgently needed for development of superior hybrids to mitigate malnutrition and to intensify maize production and productivity in sub‐Saharan Africa (SSA). This study was designed to identify early‐maturing inbred lines with combined tolerance to drought, elevated tryptophan, and PVA contents; to examine inbred–hybrid relationships for tryptophan and PVA accumulation; and to select hybrids with outstanding grain yield (GY) performance. A total of 64 inbred lines and six checks, plus 96 hybrids and four checks, were evaluated under drought and well‐watered environments in Nigeria for 2 yr. Eighteen parental lines and 54 derived hybrids were assayed for tryptophan and PVA contents. Ten drought‐tolerant inbred lines with high tryptophan and elevated PVA levels were identified in the top 10 hybrid combinations across managed drought and well‐watered conditions. The inbred–hybrid relationship was significant for GY under each and across the two contrasting environments. Significant average heterosis was found for tryptophan and PVA under well‐watered conditions. This indicated that the selected inbred lines could be used for developing high‐yielding PVA‐QPM hybrids tolerant to drought stress in SSA. The 10 top‐performing PVA‐QPM hybrids identified are being extensively evaluated in different locations and subsequently in on‐farm trials for commercialization throughout SSA.

**Abbreviations**
ASI: anthesis–silking interval
BV: breeding value
DA: days to 50% anthesis
DS: days to 50% silking
DW: dry weight
EASP: ear aspect
GY: grain yield
LD: leaf death
MPH: mid‐parent heterosis
PASP: plant aspect
PLHT: plant height
EHT: ear height
PVA: provitamin A
QPM: quality protein maize
SSA: sub‐Saharan Africa
WAP: weeks after planting
Rcond: research condition.

## 1 INTRODUCTION

Maize (*Zea mays* L.) production and productivity in sub‐Saharan Africa (SSA) is challenged by recurrent drought, which is a common yield reduction factor in the savanna agro‐ecologies of the subregion (Cairns et al., 2013). Grain yield losses can vary from 40 to 90% under drought environments (Menkir & Akintunde, 2001) in SSA. The increased rate of drought occurrence in maize production zones of the subregion significantly contributes to low grain yield (GY), which rarely exceeds 1.7 t ha^−1^ annually (FAOSTAT, 2016). Moreover, maize is often cultivated by small‐scale farmers who are unable to afford irrigation. Interventions from governments and non‐governmental organizations to provide irrigation facilities for low‐input farmers have not been sustainable due to cost implications. In view of this, producers should not rely on seasonal weather patterns to provide water to the crop because extended periods of drought can reduce yield. However, improvement in the genetic ability of maize to tolerate drought environments has been identified as the most effective and sustainable means to significantly increase genetic gains in GY from selection (Abdulmalik et al., 2017; Badu‐Apraku, Ifie, Talabi, Obeng‐Bio, & Asiedu, 2018; Edmeades, Bolaños, Chapman, Lafitte, & Bänziger, 1999) for both small‐ and large‐scale farmers in SSA. For instance, Oyekunle and Badu‐Apraku (2013) assessed the performance of 156 early‐maturing normal endosperm maize inbreds and determined heterotic patterns of the lines as well as the association between hybrid performance and performance of the lines per se. The hybrids and their parental lines were screened in different trials under moisture stress (drought) and optimal moisture (well‐watered) conditions. Higher mid‐parent heterosis (MPH) and better parent heterosis for GY were observed in the well‐watered conditions relative to the drought conditions. Significant positive correlations were found between MPH, better parent heterosis, and GY for the two contrasting soil moisture regimes. High‐yielding and stable‐yielding drought‐tolerant hybrids were identified for commercialization in West and Central Africa. The authors concluded that drought‐tolerant maize hybrids were of crucial importance in ensuring sustainable maize production and productivity in SSA.

Protein is an important dietary requirement for humans. Due to inadequate levels of lysine and tryptophan in the endosperm, normal maize grain is deficient in protein. These two amino acids account for the differences between quality protein maize (QPM) and normal maize. The discovery of several mutants (*opaque2‐ o2, floury2‐ fl2, opaque7 ‐o7, opaque6 ‐o6*, and *floury3 ‐fl3*) of the *opaque 2* gene, which resulted in reduced levels of zein fraction but elevated levels of other protein classes and essential amino acids (Ma & Nelson, 1975; McWhirter, 1971), triggered more research into the improvement of maize for its quality protein content. The recessive homozygous genotypes (*o2o2*) have considerably higher concentrations of tryptophan and lysine in relation to the heterozygotes (*O2o2*) or dominant homozygotes (*O2O2*) (Crow & Kermicle, 2002). The identification of *opaque‐2* modifiers (Opm) responsible for the hardness of the *o2*‐induced soft endosperm led to the breakthrough of selecting desirable QPM genotypes with kernel opaqueness of 25–50% (Babu, Agrawal, Saha, & Gupta, 2015). The QPM contains more than double the tryptophan and lysine content of normal maize (Teklewold et al., 2015; Vivek, Krivanek, Palacios‐Rojas, Twumasi‐Afriyie, & Diallo, 2008). As a result, overdependence on normal maize by children without enough dietary protein could lead to childhood growth failure, including “kwashiorkor,” a fatal syndrome resulting from stunted growth (Gunaratna, De Groote, Nestel, Pixley, & McCabe, 2010). The QPM can potentially provide about 90% protein quality as percentage of milk, compared with approximately 39% in normal maize (Vivek et al., 2008).

**Core Ideas**

- Drought‐tolerant PVA‐QPM hybrids are invaluable for human nutrition.
- Ten PVA‐QPM lines with high heterosis selected under drought for hybrid production.
- Strong inbred–hybrid relationships aid selection of superior PVA‐QPM hybrids.
- Ten outstanding drought‐tolerant PVA‐QPM hybrids identified for commercialization.

Furthermore, humans do not synthesize vitamin A in the body and thus rely on external sources to meet dietary requirements. Deficiency of vitamin A could lead to serious health disorders, including night blindness in children under 5 yr (Imdad, Herzer, Mayo‐Wilson, Yakoob, & Bhutta, 2010). Breeding for elevated levels of provitamin A (PVA) in maize (a major staple food in SSA) endosperm was therefore necessary to ensure that maize becomes a cheap and sustainable source of vitamin A for the millions of people in SSA who depend on the crop for their daily calories. Several studies have elucidated the breeding strategies for biofortifying maize with elevated levels of PVA, and interesting findings have been reported. For instance, Harjes et al. (2008) reported positive but weak correlation between PVA levels and orange kernel color. Similarly, Azmach, Gedil, Menkir, and Spillane (2013) observed weak or no correlations between orange kernel color and PVA. The implication of these results is that depending on orange kernel color per se to determine the PVA levels of maize genotypes could be unreliable (Menkir & Maziya‐Dixon, 2004; Menkir, Liu, White, Maziya‐Dixon, & Rocheford, 2008). It is therefore necessary to routinely conduct chemical analysis to determine the levels of PVA carotenoids in the development of hybrid maize varieties for commercialization. Extensive studies have also identified marker‐assisted selection to accelerate the detection and validation of PVA candidate genes, including *β‐carotene hydroxylase1* (*crtRB1*), *lycopene epsilon cyclase* (*LcyE*), and *phytoene synthase1* (*PSY1*), which are involved in the regulation of PVA accumulation in the PVA biosynthetic pathway (Babu, Rojas, Gao, Yan, & Pixley, 2013; Harjes et al., 2008; Obeng‐Bio et al., 2019; Owens et al., 2014; Wong, Lambert, Wurtzel, & Rocheford, 2004; Wurtzel, Cuttriss, & Vallabhaneni, 2012; Yan, Kandianis, Harjes, & Bai, 2010). The alleles of the *crtRB1* gene (crtRB1‐5′TE and crtRB1‐3′TE) have been found to be the most favorable for PVA carotenoid accumulation in both tropical and temperate maize (Babu et al., 2013; Obeng‐Bio et al., 2019). The PVA maize can potentially provide higher than the daily dietary requirement of about 15.0 μg g^−1^ dry weight (DW) PVA for human beings relative to approximately 2.0 μg g^−1^ DW found in normal yellow maize cultivars (Pixley et al., 2013).

The unique potential of maize calls for the need to develop superior drought‐tolerant orange endosperm hybrids with enhanced levels of lysine and tryptophan as well as PVA not only to ensure food security but also to provide nutrient security, especially among children and women in SSA. To address this need, the International Institute of Tropical Agriculture Maize Improvement Program (IITA‐MIP) has developed PVA inbred lines (from 2007 to 2015) that differ in their responses to limited soil moisture conditions, coupled with elevated levels of tryptophan and lysine. This has been necessary because successful performance of maize hybrids is largely associated with the inherent genetic potentials of the available outstanding parents (Betrán, Beck, Bänziger, & Edmeades, 2003). Information on inbred and hybrid genetic relationships is crucial to minimize time and cost involved in the production and evaluation of hybrids. Such information would offer the possibility to develop productive hybrids out of the myriads of hybrid combinations generated from the inbred lines available in a breeding program. Positive and significant correlation of GY between MPH of hybrid and parental lines has been reported (Betrán et al., 2003; Gissa, Zelleke, Labuschagne, Hussien, & Singh, 2007; Makumbi, Betràn, Bänziger, & Ribaut, 2011; Oyekunle & Badu‐Apraku, 2013; Wegary, Vivek, & Labuschagne, 2013; Zeleke, 2015). Therefore, maize breeders need to consider the inbred–hybrid relationship while developing drought‐tolerant hybrids under drought and optimal growing conditions. However, there is a dearth of information on the parent–hybrid relationship in the available PVA‐QPM inbred lines of the IITA‐MIP.

In the present study, the PVA‐QPM inbreds were screened with the aid of a light box to select desirable kernels (i.e., kernels that had deep orange color and endosperm opaqueness ranging from 25 to 50% for high PVA and tryptophan and lysine contents). It was therefore imperative that the PVA‐QPM inbred lines be assessed under drought conditions and that the tryptophan and PVA contents of the inbred lines be determined with the use of chemical analysis as a basis for the development of PVA‐QPM drought‐tolerant hybrids in SSA. The objectives of the present study were (a) to identify PVA inbred lines combining drought tolerance and elevated levels of tryptophan, (b) to examine the relationship between the inbred lines and hybrids for increased levels of PVA and tryptophan under optimal growing environments, and (b) to select hybrids with outstanding GY performance across drought and optimal environments.

## 2 MATERIALS AND METHODS

### 2.1 Plant materials

The inbreds used for this study were derived from the QPM variety 2009 TZE‐OR2 DT STR QPM characterized by early maturity (90–95 d to maturity), high PVA, and quality protein content of the grains. The development of this highly diverse, open‐pollinated variety commenced in 2007 with a cross between the drought‐tolerant and *Striga*‐resistant, early‐maturing QPM open‐pollinated variety TZE‐Y‐Pop‐DT‐STR‐QPM, which is characterized by mixed yellow and deep orange endosperm color, and a medium‐maturing (105–110 d to maturity) variety [Syn‐KU1409/DE3/1409‐(OR2)] with elevated PVA content from the IITA‐MIP. This presumably led to the introgression of genes coding for elevated β‐carotene into the existing QPM variety. Subsequently, a cycle of backcrossing was performed with the TZE‐Y‐Pop‐DT‐STR‐QPM parent to recover earliness. The BC_1_F_1_ lines possessing deep orange color as well as desirable endosperm modifications were identified and advanced in 2008 to obtain the F_2_ and F_3_ generations. Desirable F_3_ lines were identified based on their responses to *Striga* infestation in 2009 and were recombined to constitute the early PVA‐QPM open‐pollinated variety 2009 TZE‐OR2‐DT‐STR‐QPM. Between the years 2011 and 2014, the first generation of the early‐maturing inbred lines was extracted from the source variety, followed by series of inbreeding to the S_6_ generation using the pedigree selection method. That is, S_1_ lines were initially generated by selfing individual plants (about 1,000 plants) of the 2009 TZE‐OR2‐DT‐STR‐QPM variety. The S_1_ seeds were planted, and about 80% agronomically desirable S_1_ plants were selected to generate S_2_ seeds. The cycles of inbreeding continued until the S_6_ generation. In the course of the inbreeding program, the inbred lines were screened at the S_2_ to the S_5_ generations to select for kernels with deep orange color for high PVA and the desirable endosperm modification varying from 25 to 50% opaqueness for high tryptophan or lysine concentration. Based on the opaqueness and deep orange color of kernels, a set of 73 early‐maturing PVA‐QPM inbreds was constituted for this study. Other studies have focused on the genetic analysis of GY and other important traits for *Striga* resistance and low N tolerance (Badu‐Apraku & Fakorede, 2017) as well as validation of PVA candidate genes (Obeng‐Bio et al., 2019) of this set of inbred lines. In the present study, the GY performance of the inbred lines and their derived hybrids was assessed under drought and well‐watered environments. Additionally, the tryptophan and PVA contents of the inbred lines and hybrids were assessed under well‐watered conditions. Out of the 73 inbred lines, 64 lines, along with six inbred checks (orange endosperm inbreds found to be drought tolerant in previous studies), were evaluated under drought at Ikenne and Kadawa and under well‐watered environments at Ikenne, Nigeria. Furthermore, 24 orange endosperm inbred lines (selected based on their endosperm opaqueness ranging from 25 to 50% under the ultra‐violet light box) were crossed to obtain 96 single‐cross orange endosperm hybrids using the North Carolina Design II. The hybrids plus four checks (hybrids released and commercialized in Nigeria, Ghana, and Mali) were also evaluated under drought and well‐watered environments for 2 yr at Ikenne, Nigeria.

### 2.2 Field trials

The 70 PVA‐QPM inbred lines were evaluated under managed drought at Ikenne (6^о^50′ N, 30^о^45′ E; 62 m asl; 1,200 mm mean rainfall annually) during 2017/2018 dry season and Kadawa (11°45′ N, 8°45′ E, 468.5 m above sea level, 884 mm annual rainfall) during the 2018/2019 dry season. On the other hand, the 100 hybrids were evaluated under managed drought conditions at Ikenne during the dry seasons of 2016/2017 and 2017/2018. Drought condition was imposed by weekly application of 17 mm of sprinkler irrigation water until 25 d after planting, when the irrigation was suspended for the maize plants to depend on the residual soil moisture to complete growth and reproduction. For the trials under induced drought conditions, N–P–K (15–15–15) fertilizer was applied during planting at 60 kg ha^−1^ each for N, P, and K. Also, 30 kg ha^−1^ of N was applied as top‐dressing at 3 wk after planting (WAP) using urea fertilizer.

The inbred lines and hybrids were planted under well‐watered conditions at Ikenne during the 2016 and 2017 rainy seasons. Under optimal environments, N–P–K (15–15–15) fertilizer was applied to the inbred lines at 60 kg ha^−1^ each for N, P, and K immediately after thinning (2 WAP). At 4 WAP, top‐dressing was carried out by applying urea fertilizer at 30 kg ha^−1^ of N. The experimental plots of inbred lines and hybrids were laid out using 7 by 10 and 10 by 10 lattice designs, respectively, each replicated twice. Inbred and hybrid fields were adjacent to each other. Each plot comprised a single 4‐m‐long row, with 0.75 m inter‐row and 0.40 m intra‐row spacing. Two plants were established per hill to obtain approximately 66,667 plants ha^−1^. Pre‐ and postemergence herbicides (atrazine and gramozone, respectively) were applied at 5 l ha^−1^ to manage weeds as necessary.

### 2.3 Data collection

Based on individual plots for both inbred line and hybrid trials, data were taken for days to 50% anthesis (DA) and silking (DS) as well as plant and ear heights (PLHT, EHT). Plant aspect (PASP) and ear aspect (EASP) were rated on a scale of 1 to 9 (1 = excellent plants or ears; 9 = extremely poor plants or ears). The anthesis–silking interval (ASI) was estimated as the difference between DS and DA. At 70 d after planting, visual rating for leaf death (LD) was done for the trials conducted under induced drought using a 1–9 scale, where 1 indicates <10% of leaf death, and 9 indicates >90% of leaf death. The number of ears per plant was calculated as the number of ears harvested per plot divided by the number of all plants in the plot. Grain weight per plot was determined after shelling the harvested ears under drought environments. Moisture content of grain was measured with the aid of Kett moisture tester PM‐450. Grain weight adjusted to 15% moisture content was used to estimate GY (kg ha^−1^) per plot. For the trials conducted under optimal growing conditions, 80% shelling percentage was assumed for computing GY based on ear weight adjusted to 15% moisture content.

### 2.4 Kernel samples production for quantification of tryptophan and provitamin A contents

Seventy‐three genotypes including 54 hybrids, 18 inbred lines and a QPM standard check (Obatanpa) were selected to produce kernel samples for analysis of tryptophan and PVA contents. The 54 hybrids were selected from the 96 derived hybrids based on GY performance. The 18 inbred lines were parents involved in generating the 54 hybrids using the North Carolina design II mating arrangement. The genotypes were planted under well‐watered conditions in 2018 at IITA‐Ibadan (7°28′11.99″ N, 3°53′2.88″ E; 190 m asl) using 1‐m‐long single rows with 0.75 m between rows and 0.20 m within a row. One plant per hill was established to provide five plants per plot. Maize grain samples of hybrids and inbred lines were produced by self‐pollination of individual plants in each plot. Ears harvested were dried to 12% moisture content at room temperature, and individual ears were shelled separately. Sixty kernels were taken from each ear (one ear per plant) to form a bulk representative sample of 300 kernels. Thereafter, 100 kernels were drawn from each genotype for PVA and tryptophan analyses using high‐performance liquid chromatography (Howe & Tanumihardjo, 2006) and the colorimetric technique (Herbabdes & Bates, 1969), respectively, in the Chemical Laboratory of IITA, Ibadan, Nigeria. However, because Obatanpa is an open‐pollinated variety, the 100 kernels were drawn from a bulk of seeds harvested from an isolated field that consisted of over 1,000 plants. For quality protein levels, only tryptophan content was analyzed in endosperm flour. This is because tryptophan content has high correlation with that of lysine (*r* > .9) (Nurit, Tiessen, Pixley, & Palacios‐Rojas, 2009). Moreover, tryptophan quantification is cheaper relative to lysine. Therefore, maize breeders of West and Central Africa usually estimate tryptophan content as a measure of the quality protein status of QPM genotypes, especially during the initial stages of the breeding schemes.

### 2.5 Statistical analysis

Data measured on the inbred lines and derived hybrids were used in ANOVA under each and across environments using the general linear procedure (PROC GLM) in Statistical Analysis Software, with a random statement and a test option (SAS, 2012). In the ANOVA, the location and year combinations constituted an environment, and the optimal and induced drought environments were regarded as research conditions. Environments, replicates within environments, and incomplete blocks within the interactions of replicates × environment were made random factors, whereas inbred lines and hybrids were made fixed factors in the statistical model. Significant differences among means were detected using standard error of difference (SED). Best linear unbiased predictions, also referred to as “predicted breeding values,” were computed for PVA content of the inbred lines using the ‘lmerTest’ package in the R statistical software (R Core Team, 2013). The inbred lines were set as random factors, and the model was fitted using the ‘lmer’ function in R. Predicted breeding values for PVA content were computed using the ‘ranef’ function in R. Variance components due to genotype and phenotype of the inbred lines and hybrids were computed on genotype mean basis for the separate research conditions and across research conditions using the restricted maximum likelihood method. The variance component procedure (PROC Varcomp) implemented in SAS was applied to compute repeatability (R) for the measured traits as follows:

$$R\;=\;\frac{\sigma_{g}^{2}}{\sigma_{g}^{2}+\frac{\sigma_{\mathrm{ge}}^{2}}{e}+\frac{\sigma^{2}}{re}}$$

where σ_g_^2^ is the additive genetic variance (Hallauer, Carena, & Miranda‐Filho, 2010), σ_ge_^2^ is the genotype × environment interaction variance, σ^2^ is the variance of experimental error, *e* is number of environments, and *r* is the replicates within an environment. Identification of drought‐tolerant hybrids and inbreds was accomplished using the Multiple‐trait base Index (M.I) proposed by Badu‐Apraku, Fakorede, Oyekunle, and Akinwale (2011). The traits used in the selection index included GY, number of ears per plant (EPP), ASI, PASP, EASP, and LD under drought conditions as indicated in the following formula:

$$M.I\;=\;\left[\left(\mathrm{GY}\times \;2\right)+\mathrm{EPP}-\mathrm{ASI}-\mathrm{PASP}-\mathrm{EASP}-\mathrm{LD}\right]$$

Each trait was standardized using a SD of 1 and a mean of 0 to minimize the effects of different scales in the measurement of the different traits under drought conditions. Inbred lines and hybrids with positive M.I values were tolerant to drought, whereas those with negative values were susceptible to drought. In addition, ANOVA was performed for tryptophan and PVA data of the inbred lines and hybrids in SAS, and SED was used for the mean separation. The per se mid‐parent value for parental lines was computed as the mean of the two inbred lines averaged for each research condition and across the two environments. Mid‐parent heterosis (MPH) was estimated for the individual traits as follows:

$$\mathrm{MPH}\;=\left(\frac{F_{1}-\mathrm{MP}}{\mathrm{MP}}\right)\;\times 100$$

where F_1_ is the hybrid mean, and MP is the mean of the parental inbred lines. Average MPH were separately estimated for the traits under optimal and drought conditions and across the two contrasting research conditions. The relationship between per se mid‐parent values and MPH of corresponding hybrids were assessed for the traits under each and across the contrasting research conditions using the correlation procedure (PROC CORR) with the Spearman's rank method implemented in SAS. The difference between the mean of F_1_ hybrids and that of mid‐parents was tested for the measured traits by performing a *t* test (Wynne, Emery, & Rice, 1970) as follows;

$$t_{ij}\;=\frac{{F_{1}}_{ij}-\mathrm{MP}}{\sqrt{\frac{3}{8}\mathrm{EMS}}}$$

where F_1_ *_ij_* represents the mean of the *ij*th F_1_ cross, MP*_ij_* represents the mid parent for the *ij*th cross, and EMS represents the mean square of error.

## 3 RESULTS

Under drought conditions, ANOVA of the inbred trials revealed significant variation for environments (E) and inbred lines (G) for the measured traits except ASI and EASP for environment (Table 1). Inbred × environment interactions (GEI) were significant (*p* < .01 or *p* < .05) for the measured traits except LD. High repeatability (R) estimates were observed for the yield‐related traits compared with that of GY under drought. The R estimates obtained for ASI and plant height among the inbred lines were high under drought. Furthermore, evaluation of the inbred lines under well‐watered environments revealed significant (*p* < .05) variation for E, G, and GEI mean squares for the measured traits. The exceptions were E mean squares for ASI and E and GEI mean squares for PASP and EASP. Higher R estimates were found for most measured traits under well‐watered conditions relative to their counterparts under drought conditions. Across drought and well‐watered environments, significant variation was observed among E, research conditions (Rcond), inbred, inbred × Rcond, and GEI mean squares for the measured traits except Rcond mean squares for DA and DS. Repeatability estimates ranging from 12% for both DA and PASP to 64% for ASI were observed across research conditions.

**TABLE 1:** Mean squares and repeatability estimates of 70 early‐maturing provitamin A–quality protein maize inbreds under drought (Ikenne and Kadawa) and well‐watered (Ikenne) environments and across environments in Nigeria from 2016 rainy season to 2018/2019 dry season

Source	DF	GY	DA	DS	ASI	PLHT	PASP	EASP	EPP	LD
		kg ha^−1^				cm				
**Drought conditions**										
Env	1	14,555,495.9**	33.17*	36.50**	0.44	3,390.10**	17.90**	1.63	1.82**	38.63**
Rep (Env)	2	998,660.2**	2.89	6.46	0.83	1,322.15**	0.66	7.80*	0.02	5.16**
Block (Env × Rep)	36	201,441.4**	8.52	7.77*	2.37	228.56*	1.55**	2.56*	0.03	1.71**
Inbred	69	765,469.5**	40.41**	14.21**	19.90**	1,822.75**	3.16**	7.48**	0.24**	2.33**
Inbred × Env	69	354,381.9**	9.87*	6.69*	3.26**	265.76**	0.99*	2.67*	0.08**	0.92
Error	102	92,661.3	6.83	4.45	1.86	129.82	0.64	1.72	0.02	0.64
Repeatability, R	–	0.53	0.77	0.55	0.85	0.86	0.73	0.65	0.63	0.65
**Well‐watered conditions**										
Env	1	12,826,485.9**	48.28**	23.93*	1.92	969.53**	2.56	0.04	1.18**	–
Rep (Env)	2	605,555.6**	2.83	3.35	7.11*	203.04	7.29**	5.41*	0.14*	–
Block (Env × Rep)	36	169,686.3	11.28*	8.70**	1.72	188.04**	1.85**	2.40*	0.06*	–
Inbred	69	1,243,472.2**	45.34**	13.87**	517.64**	2,221.31**	4.50**	9.35**	0.28**	–
Inbred × Env	69	295,520.7**	10.12*	7.55*	2.86*	316.60**	0.81	1.82	0.07**	–
Error	102	111,033.2	6.82	4.57	2.25	127.09	0.84	1.49	0.03	–
Repeatability, R	–	0.75	0.79	0.50	0.84	0.87	0.82	0.84	0.77	–
**Across drought and well‐watered conditions**										
Environment	3	10,173,792.6**	34.24**	20.92**	8.58**	1,992.40**	8.37**	8.66**	1.22**	
kRcond	1	3,139,396.2**	21.28	2.34	23.38**	1,617.56**	4.63*	24.31**	0.65**	
Rep (Env)	4	802,107.9**	2.86	4.90	3.97*	762.60**	3.97**	6.61*	0.08*	
Block (Env × Rep)	72	185,563.8**	9.90*	8.23**	2.04	208.30**	1.70**	2.48**	0.04*	
Inbred	69	745,141**	26.33**	18.20**	12.76**	1,218.93**	2.37**	5.47**	0.19**	
Inbred × Rcond	69	158,5521.4**	58.98**	8.99*	32.15**	3,074.44**	5.28**	11.94**	0.42**	
Inbred x× Env	207	333,695.1**	23.57**	7.74**	4.53**	803.34**	2.07**	4.43**	0.09**	
Error	204	101,847.2	6.82	4.51	2.06	128.46	0.74	1.61	0.03	
Repeatability, R	–	0.55	0.12	0.57	0.64	0.34	0.12	0.19	0.52	

*Note*. ASI, anthesis–silking interval; DA, days to 50% anthesis; DS, days to 50% silking; EASP, ear aspect (1–9); Env, environment; EPP, ears per plant; GY, grain yield; LD, leaf death (1–9); PASP, plant aspect (1–9); PLHT, plant height; Rcond, research condition.

^*^Significant at the .05 probability level. ^**^Significant at the .01 probability level.

Analysis of variance of the hybrid data revealed significant variation among E, G, and GEI mean squares for most traits under each environment and across environments (Tables 2 and 3). The R values estimated for GY of hybrids were consistently high under moisture stress, under optimal moisture, and across environments. Forty‐six percent of the inbred lines showed tolerance to drought, signalling their usefulness as an invaluable source of drought‐tolerant alleles for the development of drought‐tolerant hybrids and synthetic varieties. Comparing the mean GY of the inbred lines for the drought trials with that under well‐watered conditions revealed high variation in yield reductions from 16% for TZEIORQ 47 to 91.5% for TZEIORQ 32 (average, 59%) (Table 4). Generally, inbred lines with high and positive selection indices recorded lower ASI under drought environments.

**TABLE 2:** Mean squares and repeatability estimates of 100 early‐maturing provitamin A–quality protein maize hybrids evaluated under drought and well‐watered environments at Ikenne, Nigeria, from 2016 rainy season to 2017/2018 dry season

Source	DF	GY	DA	DS	ASI	PLHT	PASP	EASP	EPP	LD
		kg ha^−1^				cm				
**Drought conditions**										
Env	1	278,571,020.4**	22,535.68**	21,928.04**	4.15	3,722.39**	38.34**	162.87**	3.54**	224.20**
Set	5	2,553,717.4*	5.37*	4.67	6.65**	345.38*	1.33*	1.85*	0.07*	1.09*
Env × Set	5	1,028,828.8	11.64**	13.26**	0.62	636.49**	1.39*	0.91	0.08	0.87*
Rep (Env × set)	10	706,104.1	3.44	4.93	2.63	72.04	0.38	0.84	0.04	0.41
Block (Env × rep)	36	1,102,824.7	5.18**	9.36**	3.28*	709.79**	1.33**	1.10*	0.03	0.73**
Hybrid	99	7,678,135.4**	15.63**	23.90**	5.05**	1,100.55**	2.89**	6.78**	0.20**	1.43**
Male (Set)	18	9,683,257.3**	17.48**	23.93**	3.41*	901.65**	1.79**	6.27**	0.15**	0.99**
Female (Set)	18	6,861,305.6**	9.24**	12.48**	4.98**	1,471.45**	2.66**	5.95**	0.19**	2.47**
Female × male (Set)	54	8,068,468.2**	18.10**	28.89**	5.45**	1,137.85**	3.57**	7.88**	0.24**	1.11**
Hybrid × Env	99	1,943,928.1**	11.94**	11.62**	3.20**	264.96**	0.70*	1.04*	0.06**	0.91**
Env × male (Set)	18	3,080,980.5**	16.35**	14.56**	5.57**	171.4*	0.7*	1.60**	0.06*	1.57**
Env × female (Set)	18	2,343,428.5**	10.30**	11.29**	4.70**	346.25**	0.96*	1.11*	0.11**	1.31**
Env × female × male (Set)	54	1,571,630.6*	11.45**	10.77**	2.21*	247.12*	0.54	0.75	0.05*	0.54*
Error	144	973,235	1.90	3.36	1.94	156	0.48	0.73	0.04	0.35
Repeatability, R		0.75	0.25	0.51	0.36	0.78	0.78	0.85	0.70	0.38
**Well‐watered conditions**										
Env	1	410,368,458.0**	124.53**	175.31**	9.99**	5,450.85**	41.54**	139.33**	0.87**	–
Set	5	3,854,918.5**	5.36**	3.44*	0.73	1,692.08**	1.25*	2.12**	0.04	–
Env × Set	5	712,721.0	4.44**	3.50*	0.84	716.06**	0.95	0.25	0.06*	–
Rep (Env × Set)	10	1,319,248.3	1.33	2.14	0.33	53.99	0.55	0.78	0.01	–
Block (Env × Rep)	36	1,362,458.9	1.70*	2.21**	0.37	273.97**	0.71*	0.85**	0.02	–
Hybrid	99	10,917,320.0**	5.98**	7.28**	1.14**	1,292.23**	1.95**	5.18**	0.03**	
Male (Set)	18	8,999,701.7**	5.94**	6.23**	0.87*	1,420.97**	2.21**	5.15**	0.05**	–
Female (Set)	18	11,754,081.5**	9.16**	9.50**	1.56**	2,181.14**	1.61**	5.08**	0.03	–
Female × male (Set)	54	12,577,802.5**	4.64**	7.18**	1.14**	955.71**	2.19**	6.05**	0.03	–
Hybrid × Env	99	2,6242,86.0**	2.72**	2.93**	0.65	308.39**	0.64*	0.86**	0.02*	–
Env × male (Set)	18	2,312,319.5**	2.58**	2.70**	0.60	269.22**	0.43	0.71*	0.02	–
Env × female (Set)	18	3,006,259.9**	3.27**	3.25**	0.65	372.14**	0.59	0.78*	0.03	–
Env × female × male (Set)	54	2,910,861.0**	2.64**	3.08**	0.62	278.27**	0.70**	1.11**	0.02	–
Error	144	1,084,199	1.00	1.22	0.52	89.53	0.43	0.42	0.02	–
Repeatability, R		0.77	0.53	0.59	0.42	0.77	0.74	0.87	0.31	–

*Note*. ASI, anthesis–silking interval; DA, days to 50% anthesis; DS, days to 50% silking; EASP, ear aspect (1–9); Env, environment; EPP, ears per plant; GY, grain yield; LD, leaf death (1–9); PASP, plant aspect (1–9); PLHT, plant height.

^*^Significant at the .05 probability level. ^**^Significant at the .01 probability level.

**TABLE 3:** Mean squares and repeatability estimates of 100 early‐maturing provitamin A–quality protein hybrids across drought and well‐watered environments at Ikenne, Nigeria, 2016–2018

Source	DF	GY	DA	DS	ASI	PLHT	PASP	EASP	EPP
		kg ha^−1^				cm			
Env	3	672,591,693.0**	8,256.42**	9,084.38**	214.29**	50,008.50**	29.96**	127.54**	2.56**
Set	5	4,758,404.0**	7.05**	5.90*	2.59	16,18.31**	2.58**	2.28**	0.15**
Env × set	15	1,074,726.0	6.59**	6.34**	2.18*	611.48**	0.85*	0.94	0.06*
Rep (Env × set)	20	1,012,666.0	2.39*	3.54	1.48	63.01	0.47	0.81	0.03
Block (Env × Rep)	72	1,232,641	3.44**	5.78**	1.83*	491.88**	1.03**	0.98**	0.03
Rcond	1	1,411,554,512.0**	2,227.78**	5,413.20**	657.03**	148,436.66**	12.50**	83.85**	4.26**
Hybrid	99	1,541,6602.0**	14.68**	22.70**	3.69**	2,041.37**	4.24**	10.23**	0.12**
Male (Set)	18	136,16,456.0**	12.21**	18.30**	2.76**	1,947.78**	3.34**	9.03**	0.07**
Female (Set)	18	13,883,452.0**	13.38**	15.12**	4.93**	3,253.51**	3.87**	9.05**	0.11**
Female × male (Set)	54	18,581,283.0**	16.29**	28.05	3.72**	1,854.75**	5.12**	12.80**	0.14**
Rcond × hybrid	99	3,080,249.0	7.74	8.89	2.32*	313.63	0.62	1.61	0.12**
Hybrid × Env	297	2,420,320.0**	7.08**	7.43**	2.06**	278.29**	0.62**	1.10**	0.07**
Env × male (Set)	54	3,455,400.0**	9.95**	9.21**	2.50**	275.59**	0.66*	1.49**	0.05**
Env × female (Set)	54	3,332,435.0**	6.20**	6.99**	2.32**	352.30**	0.71*	1.33**	0.08**
Env × female × Male (Set)	162	2072,086.0**	6.85**	7.27**	1.80**	228.63**	0.55	0.92**	0.07**
Error	288	1,028,716	1.46	2.29	1.23	122.77	0.55	0.58	0.03
Repeatability, R		0.84	0.51	0.67	0.47	0.88	0.87	0.89	0.40

*Note*. ASI, anthesis–silking interval; DA, days to 50% anthesis; DS, days to 50% silking; EASP, ear aspect (1–9); Env, environment; EPP, ears per plant; GY, grain yield; PASP, plant aspect (1–9); PLHT, plant height; Rcond, research condition.

^*^Significant at the .05 probability level. ^**^Significant at the .01 probability level.

**TABLE 4:** Performance of selected early provitamin A–quality protein maize inbred lines (best 10 and worst 5) plus best two checks under drought (Ikenne and Kadawa) and well‐watered (Ikenne) environments and across environments in Nigeria from 2016 rainy season to 2018/2019 dry season

	Grain yield				ASI		Plant aspect		Ear aspect		Ears per plant		Leaf	
Inbred line	Drought	Optimal	Across Env	Yield reduction	Drought	Optimal	Drought	Optimal	Drought	Optimal	Drought	Optimal	Deaf	M.I
	kg ha^−1^			%										
TZEIORQ 23	790	1,221	1,006	35.3	3.0	−1.0	3.9	3.9	4.2	4.9	0.4	0.8	3.4	7.3
TZEIORQ 5	564	1,069	817	47.3	3.0	1.0	4.9	4.2	4.9	5.1	0.6	0.9	3.6	4.8
TZEIORQ 29	552	1,112	832	50.4	3.0	1.0	4.7	4.3	4.2	7.9	0.6	0.6	3.6	4.8
TZEIORQ 26	448	1,192	720	62.5	3.0	1.0	3.5	4.5	3.2	5.7	0.4	0.6	3.7	4.7
TZEIORQ 7	564	1,134	849	50.3	3.0	0.0	4.8	4.5	4.4	4.7	0.5	0.7	3.8	4.6
TZEIORQ 6	468	1,139	804	58.9	3.0	0.0	4.0	3.8	5.0	4.4	0.6	0.8	3.7	4.1
TZEIORQ 47	444	529	487	16.1	5.0	1.0	3.4	4.2	4.5	6.8	0.6	0.5	4.3	3.3
TZEIORQ 20	419	693	506	39.5	4.0	0.0	4.1	4.9	5.3	6.4	0.4	0.6	4.1	1.9
TZEIORQ 24	303	477	390	36.6	4.0	0.0	5.1	4.8	5.3	4.9	0.6	0.8	3.9	0.8
TZEIORQ 42	347	918	633	62.2	4.0	1.0	4.9	4.5	4.8	6.4	0.5	0.6	4.3	0.6
TZEIORQ 52	188	973	393	80.7	4.0	0.0	5.5	4.8	7.6	4.8	0.1	0.7	5.0	−6.0
TZEIORQ 46	165	434	300	62.0	5.0	1.0	5.9	4.9	6.8	5.6	0.3	0.4	6.7	−7.2
TZEIORQ 10	139	1,336	738	89.5	6.0	0.0	6.2	4.3	7.8	3.9	0.2	0.9	6.0	−8.9
TZEIORQ 41	157	1,337	747	88.3	6.0	0.0	6.4	3.9	8.5	4.7	0.1	0.6	6.1	−9.9
TZEIORQ 32	128	1,512	820	91.6	7.0	0.0	6.7	2.9	8.8	4.0	0.1	0.8	5.0	−10.5
Check 1: TZEI 24	587	1,863	1,175	68.5	4.0	0.0	5.5	2.7	4.4	3.9	0.4	0.8	3.9	2.7
Check 2: TZEI 129	568	1,723	995	67.0	3.0	0.0	5.4	2.9	5.3	3.9	0.5	0.7	4.6	2.6
Mean	402	1,098	718	59.2	4.0	0.0	5.0	4.1	5.6	5.2	0.4	0.7	4.4	–
SED	64.84	1,23.78	78.85	–	0.46	0.001	0.28	0.20	0.53	0.35	0.07	0.05	0.27	–

*Note*. ASI, anthesis–silking interval; Env, environment; M. I, multiple trait base index.

Sixty‐one percent of the hybrids were identified to be tolerant to drought on the basis of the drought‐tolerant base index developed by the IITA‐MIP. Ten inbred lines (TEIORQ 29, TEIORQ 24, TEIORQ 26, TEIORQ 20, TEIORQ 6, TEIORQ 47, TEIORQ 23, TEIORQ 7, TEIORQ 5, and TEIORQ 42) that combined elevated levels of PVA and tryptophan with drought tolerance were involved in the top 10 high‐yielding PVA‐QPM hybrids evaluated under drought (Table 5). Grain yield performance of the hybrids under drought conditions varied from 348 kg ha^−1^ for TZEIORQ 13 × TZEIORQ 15 to 5,291 kg ha^−1^ for TZEIORQ 29 × TZEIORQ 24 (mean, 3,592 kg ha^−1^). The highest‐yielding PVA‐QPM hybrid (TZEIORQ 29 × TZEIORQ 24) had the lowest percentage GY reduction of 2.3, whereas the lowest‐yielding hybrid had the highest percentage GY reduction of 86. Moreover, analysis of data under drought environments generally revealed that hybrids that had reduced ASI and LD ratings and lower percentage GY reduction also had higher mean GY and positive selection indices. The best yielding PVA‐QPM hybrid out‐yielded the best commercial PVA‐hybrid check (TZEI 124 × TZEI 25) by 32%.

**TABLE 5:** Performance of early provitamin A–quality protein maize single cross hybrids (best 10 and worst 5) plus four checks under drought and well‐watered environments and across environments in Nigeria from 2016 rainy season to 2017/2018 dry season

	Grain yield				ASI		Plant aspect		Ear aspect		Ears per plant			
Hybrid	Drought	Optimal	Across aEnv	Yield reduction	Drought	Optimal	Drought	Optimal	Drought	Optimal	Drought	Optimal	Leaf death	M.I
	kg ha			%										
TZEIORQ 29 × TZEIORQ 24	5,291	5,413	5,396	2.3	1.4	0.3	4.2	4.2	2.7	4.2	0.9	1.0	3.6	9.2
TZEIORQ 24 × TZEIORQ 41	4,499	6,005	5,552	25.1	1.0	0.9	4.0	2.8	3.3	2.7	1.0	1.0	2.8	9.0
TZEIORQ 40 × TZEIORQ 26	4,853	6,025	5,439	19.5	1.5	0.2	3.8	3.5	3.3	3.6	1.0	1.1	3.3	8.7
TZEIORQ 20 × TZEIORQ 45	5,012	6,553	5,782	23.5	1.4	0.4	3.6	3.0	3.7	3.6	1.0	1.0	3.8	8.2
TZEIORQ 6 × TZEIORQ 29	4,728	5,385	5,056	12.2	0.9	0.2	4.3	3.2	3.4	4.0	1.0	1.0	3.6	8.1
TZEIORQ 26 × TZEIORQ 47	5,085	6,361	5,723	20.1	2.4	0.3	4.2	3.2	2.4	3.3	1.0	1.0	3.7	7.9
TZEIORQ 47 × TZEIORQ 23	4,166	5,699	4,932	26.9	1.7	0.4	3.9	3.6	3.7	4.1	0.9	0.9	3.2	7.1
TZEIORQ 7 × TZEIORQ 42	4,781	5,812	5,296	17.7	1.5	0.6	4.2	3.6	3.8	4.2	1.0	1.0	3.8	7.0
TZEIORQ 40 × TZEIORQ 5	4,624	5,557	5,090	16.8	1.5	0.4	3.9	3.4	3.4	4.3	0.8	0.9	3.6	6.9
TZEIORQ 5 × TZEIORQ 47	4,141	5,939	5,040	30.3	1.9	0.0	4.4	3.7	3.9	4.1	1.0	0.9	3.3	6.2
TZEIORQ 5 × TZEQI 82	2,671	6,213	4,442	57.0	3.6	0.8	5.2	2.9	4.7	3.3	0.8	0.9	3.7	−0.3
TZEIORQ 2 × TZEQI 82	2,830	7,155	4,993	60.4	4.2	1.2	4.9	2.3	4.6	2.3	0.7	1.0	4.3	−1.4
TZEQI 82 × TZEIORQ 15	1,815	5,564	3,690	67.4	3.3	1.0	5.0	3.6	6.1	4.2	0.6	1.0	4.7	−4.4
TZEIORQ 15 × TZEIORQ 11	1,635	2,663	2,149	38.6	3.4	1.4	6.2	4.1	7.5	6.2	0.4	0.8	5.7	−9.4
TZEIORQ 13 × TZEIORQ 15	348	2,519	1,434	86.2	3.9	0.5	6.8	5.1	7.6	6.6	0.2	0.9	5.7	−12.9
Check 1: TZEI 124 × TZEI 25	3,607	6,616	5,111	45.3	1.1	0.5	3.8	3.1	4.3	3.7	0.9	1.1	3.0	6.4
Check 2: TZE Pop DT STR × TZEI 17	3,147	6,399	4,773	50.8	1.9	0.6	4.9	4.3	4.7	4.0	0.8	0.9	2.9	3.6
Check 3: TZEIOR 127 × TZEIOR 57	2,749	5,886	4,318	53.3	1.5	0.5	4.5	4.3	4.3	4.2	0.8	1.0	3.7	2.8
Check 4: TZE Pop DT STR × TZEI 13	2,265	4,901	3,583	53.8	3.6	−0.2	5.3	3.4	5.3	4.4	0.6	0.9	3.7	−1.7
Mean	3,592	5,614	4,658	37.2	2.2	0.5	4.6	3.5	4.4	4.0	0.8	1.0	3.8	
SED	378.85	327.60	324.47	6.07	0.29	0.11	0.23	0.17	0.38	0.27	0.06	0.02	0.21	

*Note*. ASI, anthesis–silking interval; Env, environment; M. I., multiple trait base index.

About 90% of the inbred lines and all the hybrids assayed had more than 0.075% tryptophan per sample in whole grain (Table 6). Seventeen of the 18 inbred lines assayed for tryptophan possessed elevated levels of tryptophan, of which 12 were drought tolerant. Obatanpa, the QPM standard check, had the highest content of tryptophan. In terms of tryptophan content, Obatanpa exceeded the best inbred (TZEIORQ 42) and the best hybrid (TZEIORQ 5 × TZEIORQ 47) by 37 and 23%, respectively. Generally, high contents of tryptophan were observed for the inbred lines and hybrids (Table 6). However, low to moderate levels of PVA (compared with the average daily dietary requirement of 15.0 μg g^−1^ DW for women and children under 5 yr) were found for both inbred lines and hybrids. Predicted breeding values (BVs) of PVA computed for the inbred lines ranged from −2.30 for TZEIORQ 47 to 3.76 for TZEIORQ 29. Inbred lines with positive BVs for PVA implied that they can accelerate gains from selection to increase PVA accumulation in derived hybrids. High repeatability (R) estimates of tryptophan and PVA were detected for the inbred lines and hybrids. This indicated that the use of recurrent selection procedures could improve the accumulation of PVA and tryptophan contents of populations undergoing recurrent selection programs. Wide variation in MPH of tryptophan was observed for the selected hybrids, ranging from 0.69% for TZEIORQ 7 × TZEIORQ 42 to 84% for TZEIORQ 5 × TZEIORQ 47. However, a relatively narrow range of MPH values of PVA (6% for TZEIORQ 5 × TZEIORQ 47 to 10% for TZEIORQ 29 × TZEIORQ 24) was detected for the hybrids (Table 6).

**TABLE 6:** Tryptophan content of 18 selected early maturing provitamin A (PVA)‐quality protein maize inbreds and derived hybrids (best 15 across drought and optimal environments) along with breeding values of PVA under optimal conditions at Ibadan, Nigeria, in 2018

							MPH	
Inbred line	Tryp	PVA	BV PVA	Hybrid	Tryp	PVA	Tryp	PVA
	%	μg g^−1^			%	μg g^−1^	%	
TZEIORQ 41	0.096	7.70	−0.23	TZEIORQ 29 × TZEIORQ 24	0.131	9.84	17.49	10.34
TZEIORQ 29	0.119	12.10	3.76	TZEIORQ 24 × TZEIORQ 41	0.118	8.61	17.72	7.43
TZEIORQ 20	0.087	8.36	1.54	TZEIORQ 40 × TZEIORQ 26	0.139	7.90	41.10	8.40
TZEIORQ 42	0.124	8.38	−0.27	TZEIORQ 20 × TZEIORQ 45	0.084	6.90	12.95	7.42
TZEIORQ 70	0.120	5.11	−1.93	TZEIORQ 6 × TZEIORQ 29	0.104	9.27	12.07	9.77
TZEIORQ 24	0.104	6.60	1.17	TZEIORQ 26 × TZEIORQ 47	0.105	5.87	33.33	6.37
TZEIORQ 69	0.121	6.19	−1.07	TZEIORQ 47 × TZEIORQ 23	0.118	6.10	60.42	6.60
TZEIORQ 40	0.095	5.61	−1.43	TZEIORQ 7 × TZEIORQ 42	0.112	7.53	0.69	8.03
TZEIORQ 7	0.101	5.61	0.09	TZEIORQ 40 × TZEIORQ 5	0.130	7.44	26.41	7.94
TZEIORQ 6	0.118	5.50	0.19	TZEIORQ 5 × TZEIORQ 47	0.153	5.35	84.17	5.85
TZEIORQ 26	0.102	5.28	0.48	TZEIORQ 23 × TZEIORQ 44	0.120	6.34	32.91	6.84
TZEIORQ 5	0.110	5.18	−0.22	TZEIORQ 42 × TZEIORQ 20	0.143	6.60	35.26	7.10
TZEIORQ 45	0.106	5.10	−1.74	TZEIORQ 6 × TZEIORQ 45	0.113	6.23	1.09	6.73
TZEIORQ 23	0.091	5.06	0.38	TZEIORQ 70 × TZEIORQ 2	0.106	7.22	4.18	6.75
TZEIORQ 44	0.089	4.89	−1.86	TZEIORQ 6 × TZEIORQ 69	0.115	8.53	3.82	9.02
TZEIORQ 2	0.102	3.82	−1.21	OBATANPA (QPM check)	0.198	–	–	–
TZEIORQ 47	0.056	3.55	−2.30	–	–	–	–	–
TZEIORQ 48	0.115	3.47	−1.68	–	–	–	–	–
Mean	0.103	6.00			0.124	7.32	25.57	7.64
SED	0.006	0.67			0.010	1.4	9.09	0.51
Repeatability (R)	0.83	0.82			0.970	0.65	–	–

*Note*. BV PVA, predicted breeding values for provitamin A content; MPH, mid‐parent heterosis; PVA, provitamin A; Tryp, tryptophan.

Significant positive correlations were observed between mid‐parent values and the means of the corresponding hybrids for GY, PLHT, EASP, and LD under each environment and across environments (Table 7). Similarly, significant (*p* < .05 or *p* < .01) negative heterosis was observed for DA and DS under each environment and across environments. Significant (*p* < .05 or *p* < .01) average heterosis was also detected for tryptophan and PVA contents.

**TABLE 7:** Correlation coefficients of mid‐parent value of selected early maturing provitamin A–quality protein maize inbred lines and their hybrids, plus average heterosis under drought, optimal and across environments in Nigeria, 2016–2017/2018

	*r* Value			Average heterosis		
Trait	Drought	Optimal	Across environments	Drought	Optimal	Across environments
				%		
Grain yield, kg ha^−1^	.30**	.21*	0.26*	512.89***	234.58**	314.63**
Days to 50% anthesis	.06 (ns)^a^	.29**	.29**	−2.63*	−5.49**	−5.47**
Days to 50% silking	.04 (ns)	.29**	.29**	−5.23**	−5.40**	−6.25**
Anthesis silking interval	.02 (ns)	.04 (ns)	.11 (ns)	−38.90**	1.76 (ns)	−35.62
Plant height, cm	.45**	.46**	.50**	114.05**	35.02**	44.60**
Ear height, cm	.34**	.16 (ns)	.22*	137.19**	58.98**	71.26**
Plant aspect (1–9)^b^	.24*	.14 (ns)	.31**	10.07 (ns)	28.65**	11.00 (ns)
Ear aspect (1–9)^c^	.45**	.24*	.32**	24.78***	15.38 (ns)	−4.44 (ns)
Ears per plant	.26*	.07 (ns)	.11 (ns)	214.51***	12.27 (ns)	31.28*
Leaf death (1–9)^d^	.23*	–	–	−9.81	–	–
Tryptophan, %	–	.05 (ns)	–	–	16.98**	–
Provitamin A, μg g^−1^	–	.69**	–	–	27.42*	–

^a^ Not significant at .05 probability level;

^b^ Plant aspect (on a scale of 1–9), where 1 = excellent overall phenotypic appeal and 9 = poor overall phenotypic appeal.

^c^ Ear aspect (on a scale of 1–9), where 1 = clean, uniform, large, and well‐filled ears, and 9 = rotten, variable, small, and partially filled ears.

^d^ Leaf death (on a scale of 1–9), where 1 = <10% overall dead leaf area, and 9 = >90% overall dead leaf area.

^*^Significant at the .05 probability level. ^**^Significant at the .01 probability level.

## 4 DISCUSSION

The highly significant differences in the inbred lines observed for all nine traits measured across the two contrasting research conditions implied the presence of genetic variation in the early‐maturing PVA‐QPM inbred lines, which is desirable for hybrid production. The significant inbred × environment interactions detected for the measured traits explained the inconsistent reactions of the inbred lines with respect to the traits measured under the contrasting environments and that evaluation in more environments would be necessary in identifying outstanding lines (Anley, 2013; Edmeades et al., 2013). The high repeatability (R) values estimated for the yield related traits, especially ASI and plant height, in relation to that of yield under managed drought conditions indicated that these traits could be relied on in addition to yield for the selection of outstanding drought‐tolerant inbred lines (Bänziger, Edmeades, Beck, & Bellon, 2000). The results implied that, for this set of inbred lines, ASI and plant height are stable traits that could be transferred to hybrids even under limited soil moisture conditions. Hybrids with low and stable ASI under drought would consistently have fewer days between anthesis and silking under drought for efficient pollination, fertilization, and seed set. Moreover, inheritance of good plant height (relatively taller plants) in hybrids indicates an advantage of producing enough assimilates for partitioning into sinks (grains), which could contribute to high GY. The high R estimate observed for ASI under low soil moisture environments confirmed the results of other researchers (Bänziger et al., 2000; Magorokosho & Tongoona, 2003; Santos et al., 2020), who determined that ASI is a predictable secondary trait for identifying drought‐tolerant maize genotypes. It was striking to find higher R estimates for most traits under optimal growing conditions relative to their counterparts under drought. This result indicated the existence of maximum potentials of the inbreds in the absence of drought stress and that selecting for drought tolerance would be most successful when the experiments are conducted under drought conditions (Edmeades et al., 1999, 2013) to expose the inherent responsive potentials of the genotypes to drought. Across drought and well‐watered environments, the variation due to environment, research conditions, inbred, inbred × research, and inbred × environment interactions were significant for most of the measured traits. This observation suggested that the two research conditions were very discriminating in identifying genetic variation in the set of inbreds evaluated. Relative to drought and well‐watered conditions, low R estimates ranging from 12% for DA and PASP to 64% for ASI were observed across research conditions, suggesting that, when conducting drought experiments, ASI should be regarded as a key trait among the set of traits measured to identify outstanding drought‐tolerant maize (Badu‐Apraku et al., 2016; Bänziger et al., 2000), especially during early generation testing (Badu‐Apraku, Oyekunle, Akinwale, & Aderounmu, 2013). The results revealed a wide range of yield reductions (16–91.52%), indicating that the response of the lines to the drought conditions was regulated by genetic factors because all the lines were exposed to the same level of moisture stress. This range was higher compared with the 40–90% reported by Menkir and Akintunde, (2001), an indication of wide genetic variability for drought tolerance within the PVA‐QPM inbred lines.

The significant variation observed for environment, genotype, and the genotype × environment interactions mean squares for most traits measured for the hybrids under each environment and across environments implied that there was high probability of identifying hybrids with superior performance in the set of 100 hybrids evaluated. High R values of GY were consistently estimated for the hybrids for each and across environments, indicating that selection of drought‐tolerant hybrids should be effective based on GY. Also, there is a higher possibility of transferring desirable characteristics of parental lines to hybrids. The practical implication of high R estimates is that high‐yielding hybrids under well‐watered conditions would not suffer serious yield penalties under drought. However, this result disagrees with the findings of other researchers, who found GY to be lowly heritable especially under stress and that selecting for drought tolerance using GY alone may not be successful (Bänziger et al., 2000; Tuberosa, 2012).

Sixty‐one percent of the hybrids identified to be drought tolerant in the present study confirmed the reliability of the traits used in the multiple trait base index (Badu‐Apraku et al., 2011). The 10 inbred lines that combined elevated levels of PVA and tryptophan with drought tolerance were also found to be the parents of the 10 high‐yielding PVA‐QPM hybrids under drought environments. This observation demonstrated the significance of using drought‐tolerant parents in the development of outstanding PVA‐QPM hybrids and synthetic varieties to tolerate low soil moisture conditions (Betrán et al., 2003). The best yielding PVA‐QPM hybrid (TZEIORQ 29 × TZEIORQ 24) appeared to have the lowest percentage GY reduction of 2.3, implying that this hybrid will continue to yield well under well‐watered conditions. Similar results were obtained by Bänziger et al. (2000), who observed no yield penalties under well‐watered conditions for the drought‐tolerant maize cultivars previously identified under managed drought environments. Generally, the hybrids that recorded high GY and positive selection indices also had reduced ASI, LD, and low percentage GY reductions. The 10 top‐performing hybrids significantly yielded better (12–32%) than TZEI 124 × TZEI 25, which was the superior commercial PVA hybrid check under drought environments. The best PVA hybrid check was released and commercialized in Mali and Nigeria as Tamalaka and Sammaz 41 in 2014 and in Ghana as CSIR‐Denbea in 2015 (Badu‐Apraku & Fakorede, 2017). This implied that the 10 best‐yielding, early‐maturing PVA‐QPM hybrids would be the most desirable for maize production in moisture‐stressed areas of SSA. Extensive evaluation of these hybrids on‐farm would be required to identify potential candidates for varietal release. Apart from the superior inherent ability of these hybrids to tolerate drought environments, they possess elevated levels of PVA and tryptophan as an added advantage over the existing commercial checks.

Approximately 90% of the inbred lines and all the hybrids assayed for tryptophan concentrations had more than 0.075% in sample on a whole grain basis, suggesting that the inbreds and the hybrids met the quality criteria for classifying QPM genotypes from normal maize (Teklewold et al., 2015). The high levels of tryptophan suggest the existence of the *opaque 2* genes and its modifiers for the accumulation of tryptophan and lysine concentrations in the endosperm of the inbred lines and the hybrids (Babu et al., 2015; Crow & Kermicle, 2002). Similar inferences could be drawn for the source variety 2009 TZE‐OR2‐DT‐STR‐QPM, from which the inbred lines were extracted. The high contents of tryptophan observed for the inbred lines and hybrids suggested that QPM hybrids could be successfully developed from this set of inbred lines. However, the low to moderate levels of PVA found in both inbred lines and hybrids indicated that more breeding efforts are needed to successfully develop hybrids with the required PVA levels of ≥15.0 μg g^−1^ DW (Harjes et al., 2008; Ortiz‐Monasterio et al., 2007). In view of this, predicted BVs were computed to identify parents that could facilitate gains for PVA accumulation in derived hybrids. This was necessary because reliance on orange kernel color per se for selecting high PVA maize genotype could be ineffective due to the positive but low correlation between orange kernel color and PVA levels (Azmach et al., 2013; Harjes et al., 2008; Menkir et al., 2008). Additionally, to accelerate progress in the development of superior hybrids with elevated levels of PVA, the IITA early and extra‐early maize improvement program has concentrated efforts to consistently use the high‐performance liquid chromatography method to select parental lines with high PVA levels for hybrid combinations. However, in the present study, the range of PVA values (3.47–12.10 μg g^−1^ DW) observed surpassed the 5.00–7.80 μg g^−1^ detected by Menkir et al. (2008) when 15 tropically adapted yellow maize inbred lines were evaluated. Also, PVA values ranging from 0.06 to 17.25 μg g^−1^ (mean, 5.87 μg g^−1^) have been reported by Azmach et al. (2013) using 130 inbred lines. The inbred lines that had high positive BVs for PVA in the present study (TZEIORQ 29, TZEIORQ 20, and TZEIORQ 24) could therefore make significant contributions to PVA accumulation in their progenies. This observation was confirmed by the highest PVA level of 10 μg g^−1^ recorded for TZEIORQ 29 × TZEIORQ 24, which ranked among the top 15 hybrids across the contrasting test environments. However, the differences in PVA levels of the top three drought‐tolerant hybrids were not significant. This implies that the three hybrids should be given equal opportunities for further evaluations as far as PVA levels are concerned. Also, the high repeatability (R) estimates of tryptophan and PVA detected for the inbred lines and hybrids were an indication of the possible rapid improvement in the two nutritional traits through the application of recurrent selection methods (Coors, 1999). The results also suggested that phenotypic selection based on dark orange kernel color could be effective (Chandler et al., 2013) in this set of hybrids. However, care must be taken in using orange kernel color to select maize genotypes with high PVA concentrations because over‐reliance on this finding could be misleading (Azmach et al., 2013; Harjes et al., 2008; Menkir & Maxiya‐Dixon, 2004; Menkir et al., 2008; Obeng‐Bio et al., 2019).

The significant and positive correlations observed between the means of F_1_ hybrids and the corresponding mid‐parent means for GY, PLHT, EASP, and LD under each environment and across the two contrasting environments implied that early testing of the inbreds for the production of high‐yielding, drought‐tolerant hybrids based on these traits would be successful (Badu‐Apraku, Akinwale, Franco, & Oyekunle, 2012). Similarly, the positive and significant inbred–hybrid correlation observed for PVA indicated that inbred lines with elevated levels of PVA could transmit high PVA accumulation ability to their hybrids, an indication of the existence of additive genetic effects controlling PVA accumulation in this set of inbred lines. Several researchers have found PVA accumulation in maize to be under the influence of additive genetic effects (Egesel, Wong, Lambert, & Rocheford, 2003; Menkir, Gedil, Tanumihardjo, Adepoju, & Bossey, 2014; Owens et al., 2014; Suwarno, 2012; Suwarno, Pixley, Palacios‐Rojas, Kaeppler, & Babu, 2015), suggesting that application of recurrent selection methods should be effective (Coors, 1999). The nonsignificant correlation observed between inbred and hybrids for tryptophan content disagreed with previous findings. For instance, high tryptophan and lysine concentrations in hybrids were found to be primarily associated with the contributions of maternal inbred parents, suggesting that tryptophan and lysine contents were controlled by additive genetic effects specifically, maternal inheritance (Varadaraju & Joel, 2017). The differences in the results of the different studies may be due to the variation in the genetic materials used. Wide variation in MPH of tryptophan observed for the selected hybrids suggested that, although the hybrids were classified as QPM genotypes, significant differences existed in the ability of hybrids to accumulate elevated levels of tryptophan in their endosperm and that selection to upgrade the level of the trait would be successful. Despite the narrow range of MPH detected for PVA levels of the hybrids, the trait could be improved due to the positive PVA value observed. The significant and negative heterosis found for DA and DS for each and across the contrasting research conditions indicated that the F_1_ hybrids matured relatively earlier compared with the parental lines and that the hybrids developed vigorous and taller plants, which translated into high GY. Ofori, Ofori, Obeng‐Antwi, Tengan, and Badu‐Apraku (2015) reported similar findings under optimal conditions. Significant average heterosis recorded for tryptophan and PVA contents implied that inbreds with relatively high concentrations of tryptophan and PVA could transmit the desirable attributes to their progenies (Betrán et al., 2003). Thus, superior hybrids with respect to drought tolerance as well as tryptophan and/or PVA accumulation could be developed using this set of inbred lines.

In conclusion, 10 outstanding drought‐tolerant lines with high tryptophan and elevated PVA levels were involved in the best 10 hybrid combinations across managed drought and well‐watered conditions. The selected inbred lines would be an invaluable resource for the development of maize hybrids possessing high tryptophan levels, elevated levels of PVA, and drought tolerance for farmers in the drought‐prone production zones of SSA. The significant average heterosis obtained for plant and ear heights, DA, DS, and GY under drought indicated the high potential of developing outstanding drought‐tolerant PVA‐QPM hybrids from the 70 inbred lines studied. The 10 top‐performing PVA‐QPM hybrids identified in the present study should be widely assessed in advanced and on‐farm trials and released for intensive maize production in SSA.

## AUTHOR CONTRIBUTIONS

E. Obeng‐Bio, B. Badu‐Apraku, B.E. Ifie, A. Danquah and E.T. Blay designed this study; E. Obeng‐Bio and B. Badu‐Apraku performed the experiments; E. Obeng‐Bio and M.A. Dadzie analyzed the data; E. Obeng‐Bio and B. Badu‐Apraku drafted the manuscript; All authors revised the manuscript.

## CONFLICT OF INTEREST

The authors declare no conflict of interest.

## Data Availability

Data pertaining to this study are available to authorized users at the International Institute of Tropical Agriculture (IITA) maize improvement program data repository.
